# A novel lncRNA‐miRNA‐mRNA network analysis identified the hub lncRNA RP11‐159F24.1 in the pathogenesis of papillary thyroid cancer

**DOI:** 10.1002/cam4.1900

**Published:** 2018-11-26

**Authors:** Wei Jiang, Hua Zhan, Yanyan Jiao, Sha Li, Weixu Gao

**Affiliations:** ^1^ Department of Endocrinology the First Affiliated Hospital of Harbin Medical University Harbin China; ^2^ Department of Neurosurgery the First Affiliated Hospital of Harbin Medical University Harbin China

**Keywords:** hub gene, novel lncRNA‐miRNA‐mRNA network, papillary thyroid cancer

## Abstract

Papillary thyroid cancer (PTC) is one of the most common cancers worldwide, and its carcinogenesis is influenced by a complex network of gene interactions. In this study, the microarray expression profile was re‐annotated into a lncRNA‐mRNA biphasic profile. LncRNA‐mRNA interactions were confirmed by established miRNA‐RNA data and hypergeometric test. Then, a PTC‐related lncRNA‐miRNA‐mRNA network (PTCRN) was constructed by integrating differentially expressed genes with the RNA‐RNA networks. The new network consisted of 21 lncRNAs, 241 mRNAs and 803 edges. To prioritize PTC‐related genes, we performed topological analysis and random walk with restart (PWR) algorithm analysis of PTCRN. Both analyses identified lncRNA RP11‐159F24.1 as a hub node in the network, which could interact with 47 mRNAs by sponging miR‐485. In functional enrichment analysis, these interacting mRNAs were associated with the pathways in cancer. In validation, RP11‐159F24.1 (up‐regulated; *P* = 0.0013) showed an opposite expression pattern with its target miR‐485 (down‐regulated; *P* = 0.0013) in PTC, indicating that the RP11‐159F24.1/miR‐485/mRNAs axis might play an important role in the development of PTC. In conclusion, this study has constructed a PTC‐related lncRNA‐miRNA‐mRNA network and identified the hub lncRNA RP11‐159F24.1 in the tumorigenesis, which provided novel insights to explore the underlying mechanism of PTC.

## 1 INTRODUCTION

Thyroid cancer (TC) is one of the most common cancers around the world, among which papillary thyroid cancer (PTC) accounts for 80 ~ 90%.^1^, ^2^ Multiple factors have been identified in association with the development of PTC, like radiation exposure and thyroid nodular diseases.^3^ However, the precise mechanism remains unclear. Thus, it is essential to elucidate the molecular mechanism and develop novel and robust biomarkers and therapeutic targets for PTC.

Long noncoding RNAs (lncRNAs) are a class of RNAs with >200 nucleotides in length, which are classified into five categories (sense, antisense, bidirectional, intronic and intergenic) based on their position relative to the neighboring protein‐coding genes in the genome.^4^ LncRNAs were originally considered transcriptionally nonfunctional, but several recent studies suggested an important role of lncRNAs in PTC.^5^ In molecular mechanism, most lncRNAs could act as a microRNA (miRNA) sponge via competitive endogenous RNA (ceRNA) activity, and thus up‐regulate the expression of downstream mRNAs.^6^, ^7^ Zhang et al^8^ reported that lncRNA NEAT1 suppression inhibited PTC progression by up‐regulating miR‐129‐5p, which suppressed KLK7 expression. Wang et al^9^ found that signaling lncRNA PTCSC3/miR‐574‐5p/SCAI/Wnt/β‐catenin mediated the proliferation and migration of PTC‐1 cells. Despite of these studies, the role of lncRNA‐miRNA‐mRNA network in the pathogenesis of PTC remains elusive.

In this study, we constructed a PTC‐related lncRNA‐miRNA‐mRNA network (PTCRN) by mapping differentially expressed lncRNAs and mRNAs into an established lncRNA‐miRNA and mRNA‐miRNA network. Then, hub genes were identified by both topological analysis and random walk with a restart (RWR) algorithm analysis. Our findings could help to reveal the lncRNA‐miRNA‐mRNA regulatory network in PTC and provide novel insights in identifying the mechanism of PTC.

## 2 MATERIALS AND METHODS

### 2.1 Microarray data collection

Raw microarray data of GSE33630 were obtained from the Expression Omnibus (GEO) database (http://www.ncbi.nlm.nih.gov/geo/). The dataset was based on the chip platform of Affymetrix Human Genome U133 plus 2.0 Array (GPL570), and contained 44 pairs of PTC and adjacent normal tissues.

### 2.2 Probe re‐annotation

The microarray probe sequences were downloaded from the Affymetrix's website (http://www.affymetrix.com). The human genome (hg38) and related annotation file (Release 28) were obtained from the GENCODE database (https://www.gencodegenes.org). By using HISAT2, we identified probe‐matched lncRNA and protein‐coding sequences. Transcripts were included if fulfilling the following criteria: (a) detected by more than three probes; (b) each probe was mapped without mismatch; (c) each probe was matched to only one transcript in probe‐transcript pairs.^10^

### 2.3 Differentially expressed genes (DEGs) screening

Raw expression data were preprocessed using the “affy” R package before the probe re‐annotation. When multiple probe sets were matched to an identical gene symbol, we took the mean value to represent its average expression level. Paired *t*‐test was conducted to identify DEGs between tumor and adjacent normal tissues. A *P* value of <0.001 was considered significant.

### 2.4 Extraction of lncRNA‐miRNA and mRNA‐miRNA interactions

StarBase v2.0 (http://starbase.sysu.edu.cn) is a powerful online tool used to systematically identify RNA‐RNA and protein‐RNA interaction networks from 108 CLIP‐Seq datasets.^11^ In our study, the lncRNA‐miRNA and mRNA‐miRNA interactions were obtained from this database. Finally, we identified 10212 lncRNA‐miRNA interaction pairs (including 1127 lncRNAs and 277 miRNAs) and 423975 mRNA‐miRNA interaction pairs (including 13815 mRNA and 386 miRNAs).

### 2.5 Identification of lncRNA‐mRNA interactions with hypergeometric test

Hypergeometric test was conducted to detect competing lncRNA‐mRNA interactions and then construct the PTCRN.^11^ The significance was measured by the *P* value defined as follows:

$$P=1-\sum_{i=0}^{x-1}\frac{\left(\begin{array}{c} k\\ i \end{array}\right)\left(\begin{array}{c} T-k\\ M-i \end{array}\right)}{\left(\begin{array}{c} T\\ M \end{array}\right)}$$

where *T* was the total number of miRNAs; *M* was the number of miRNAs interacting with this given lncRNA; *k* was the number of miRNAs interacting with mRNAs; *x* was the number of miRNAs shared by the lncRNA and mRNA. A *P* value of <0.001 was considered significant.

### 2.6 Topological analysis of the PTCRN

To analyze topological features of PTCRN, the network degree, betweenness,and closeness centrality were calculated respectively by the “igraph” R package.^12^ The degree centrality (*C*_D_) represented the number of edges linked to node *v*. It was described as follows:

$$C_{\text{D}}\left(v\right)=e$$

where *e* was the number of edges linked to node *v*.

Betweenness centrality (*C*_B_) represented the number of shortest paths between all pairs of nodes in the network that passed through a specific node. It was described as follows:

$$C_{\text{B}}\left(v\right)=\sum_{s\neq t\neq v\in V}\frac{\sigma_{\text{st}}\left(v\right)}{\sigma_{\text{st}}}$$

where *σ*_st_ represented the number of the shortest path linking node *s* and node *t*, and *σ*_st_(*v*) was the node count (*v*) from node *s* to node *t*.

Closeness centrality (*C*_C_) represented the mean distance between a node and all other nodes. It was described as follows:

$$C_{\text{C}}\left(v\right)=\frac{1}{\sum_{t\in V\backslash v}^{n}\text{dist(}v\text{,}t\text{)}}$$

where dist(*v,t*) represented the average shortest paths from node *v* to all other nodes (*t*); *n* is the total number of nodes.

### 2.7 Random walk with restart (RWR) algorithm analysis of PTCRN

The RWR algorithm was a classic ranking algorithm, which simulated a random walker starting from a seed node or several seed nodes and walking on a constructed or natural network.^13^ Finally, possible novel nodes were identified and ranked from high to low probabilities. The algorithm has been adopted to search novel disease genes or other related problems.^14^, ^15^, ^16^

In this study, we selected three PTC‐related genes (STAT3, CXCL10 and SP1) as seed nodes in the RWR algorithm, which has been validated by multiple experiments. The initial probability *P*_0_ for each seed node was set as 1/*N* (where *N* was the number of seed nodes), while zero for nonseed nodes. Then, the RWR algorithm simulated a random walker that moved on the PTCRN starting from the three genes. In detail, let *P*_i_ be a vector representing the probability of each node after the *i*_th_ moving procedure was complete. After each moving procedure, *P*_i_ was updated as follows:

$$P_{\text{i + 1}}=\left(1\;\text{-}\;r\right)MP_{\text{i}}+rP_{0}$$

where *M* was the column‐wise normalized adjacency matrix of the network; *r* was the restart probability of returning to the seed nodes at every step (*r* = 0.8 in this study). When the L1‐norm of the difference between two successive vectors was <1 × 10^6^, the vector became stable, the RWR algorithm stopped and output *P*_i+1_ as the final vector. Each component in this vector indicated the probability of a node being a PTC‐related gene.

Then, we adopted a permutation test to remove the false positives, which was performed in three steps: (a) 1000 gene sets were randomly produced, and each contained three genes; (b) for each set, the RWR algorithm was executed on the PTCRN using genes in the set as seed nodes; (c) the *P* value was calculated for each gene, as follows:

$$P\left(g\right)=\frac{\theta }{1000}$$

where θ represented the number of gene sets, which yielded a higher probability of gene (*g*) than that yielded by the known PTC‐related genes. A *P* value of <0.05 was considered significant.

### 2.8 Functional enrichment analyses

To investigate potential function of candidate feature genes, gene ontology (GO) and KEGG pathway analyses were performed using The Molecular Signatures Database (MSigDB) (http://software.broadinstitute.org/gsea/msigdb) and KEGG PATHWAY Database (https://www.kegg.jp/kegg/pathway.html). False discovery rate (FDR) <0.05 was chosen as the cut‐off criteria.

### 2.9 Gene expression validation

Another GEO dataset of GSE35570 which contained 34 pairs of PTC and adjacent normal samples, was used to validate candidate lncRNA expression. To validate the lncRNA‐sponged miRNA expression, RNA‐sequencing data of 53 pairs of samples were downloaded from The Cancer Genome Atlas (TCGA) database (https://xenabrowser.net). Paired *t* test was performed, and a *P* value of <0.05 was considered significant.

## 3 RESULTS

### 3.1 Construction of PTC‐related lncRNA‐miRNA‐mRNA network

**Figure 1:**
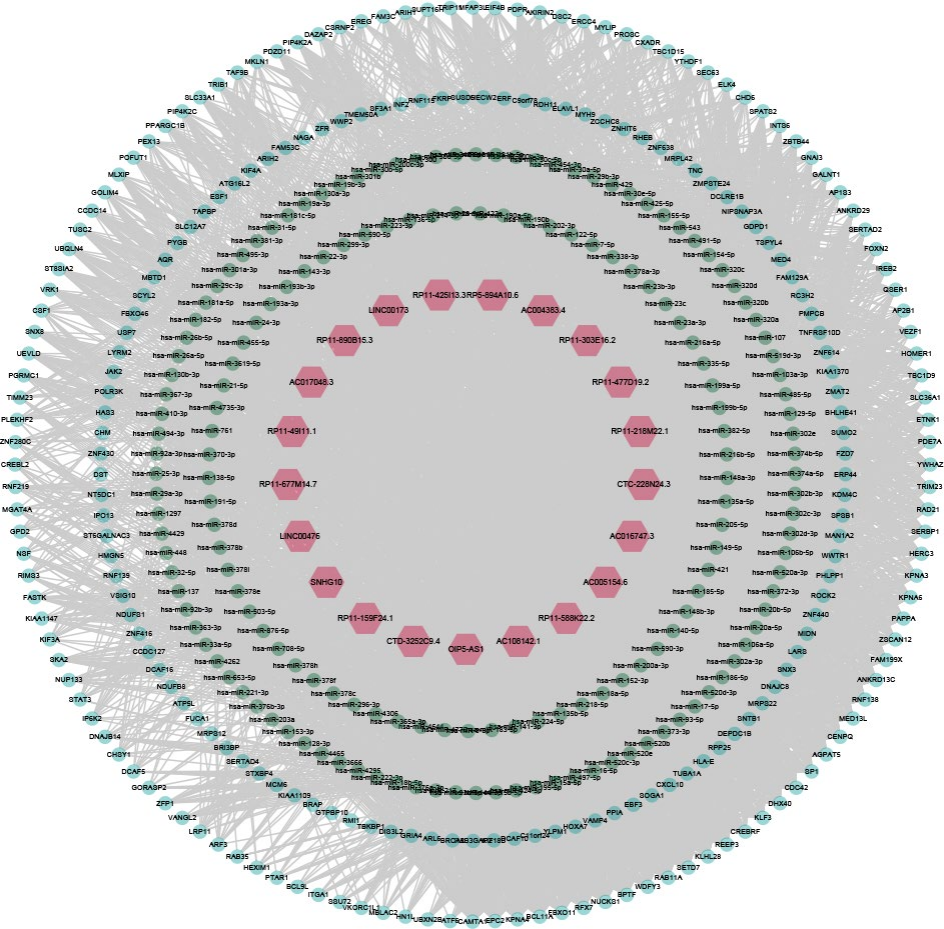
Overview of the lncRNA‐miRNA‐mRNA network related with papillary thyroid cancer. The red, green and blue nodes represented lncRNAs, miRNAs and mRNAs respectively. Gray lines represented interactions between the RNAs

After probe re‐annotation, we identified a total of 4232 lncRNAs and 11 687 mRNAs, among which 173 lncRNAs and 546 mRNAs were differentially expressed. The lncRNA‐miRNA and mRNA‐miRNA interactions from starBase v2.0 database were merged to construct the lncRNA‐miRNA‐mRNA network, in which lncRNA‐mRNA interactions were confirmed by hypergeometric test. The nodes in the network denoted lncRNAs, miRNAs and lncRNAs, while edges in the network represented the interactions between these RNAs. Finally, a PTC‐related lncRNA‐miRNA‐mRNA network (PTCRN) was constructed by integrating DEGs with the RNA‐RNA networks. The new network consisted of 21 lncRNAs, 241 mRNAs and 803 edges (Figure 1; Table S1).

### 3.2 Topological analysis of PTCRN

**Figure 2:**
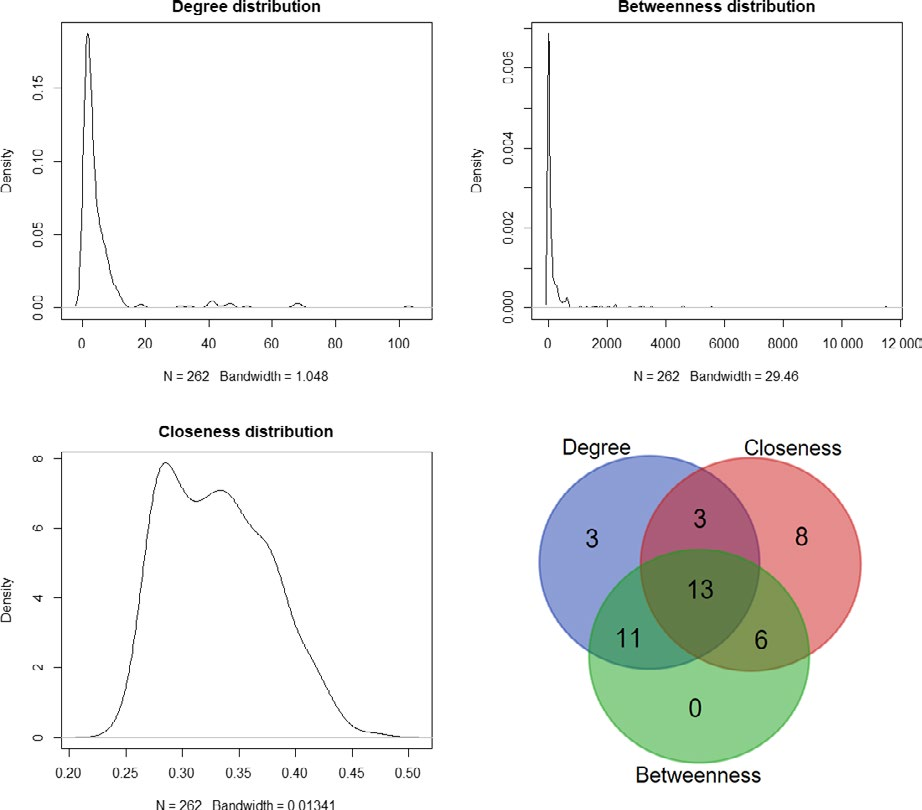
Distribution of network topological parameters. The Venn diagram showed the overlap of top 30 genes with topological features in each dimension

**Figure 3:**
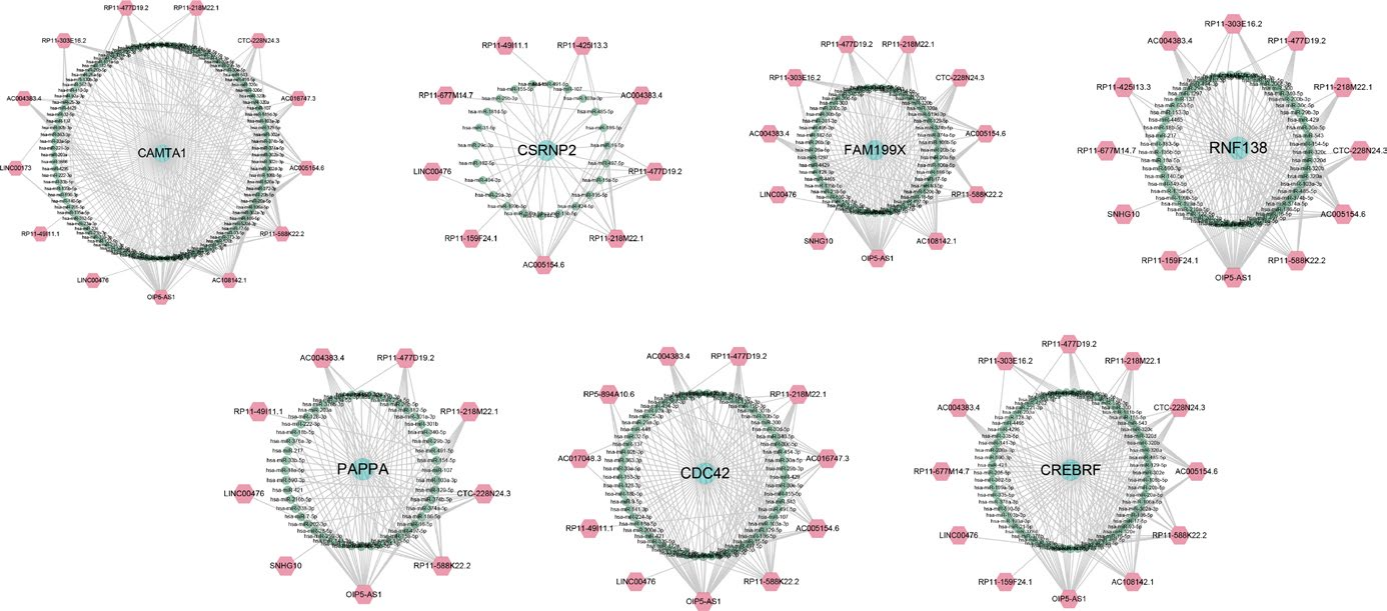
Subnetwork of seven mRNAs and their neighbors (CAMTA1, CDC42, CREBRF, CSRNP2, FAM199X, PAPPA and RNF138). The red, green and blue nodes represented lncRNAs, miRNAs and mRNAs respectively. Gray lines represented interactions between the RNAs

**Figure 4:**
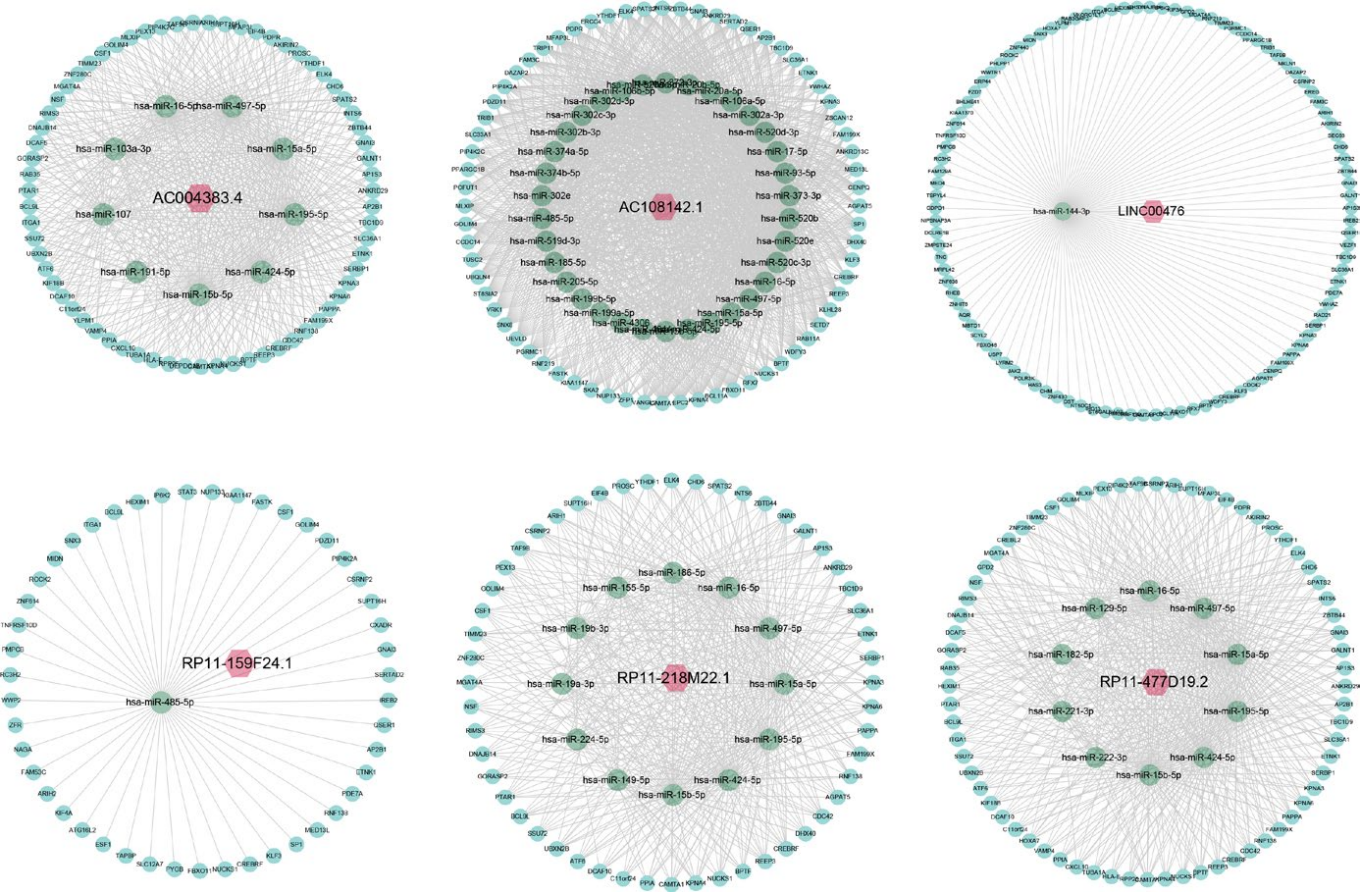
Subnetwork of five lncRNAs and their neighbors (AC004383.4, AC108142.1, LINC00476, RP11‐159F24.1, RP11‐218M22.1 and RP11‐477D19.2). The red, green and blue nodes represented lncRNAs, miRNAs and mRNAs respectively. Gray lines represented interactions between the RNAs

The topological features of the network were evaluated by the degree, betweenness and closeness centrality. We listed the overlap of top 30 genes with topological features in each dimension (Figure 2). Six lncRNAs (AC004383.4, AC108142.1, LINC00476, RP11‐159F24.1, RP11‐218M22.1 and RP11‐477D19.2) and seven mRNAs (CAMTA1, CDC42, CREBRF, CSRNP2, FAM199X, PAPPA and RNF138) were identified as hub nodes in the network (Table 1). The subnetworks of these genes and their neighbors were displayed in Figures 3 and 4.

**Table 1:** Detailed information of top 30 genes in topological analysis

Gene	Degree	Betweenness	Closeness
LINC00476	103	11514.7782	0.4686
RP11‐477D19.2	69	3222.6853	0.4176
AC108142.1	68	5564.2194	0.4149
AC004383.4	67	3123.3883	0.4149
RP11‐218M22.1	52	1524.5379	0.3961
**RP11‐159F24.1**	**47**	**2297.7300**	**0.3878**
CAMTA1	13	656.8025	0.4357
CREBRF	12	537.8600	0.4328
RNF138	12	657.5827	0.3949
CDC42	11	635.9417	0.4110
FAM199X	11	633.8818	0.4401
CSRNP2	9	498.9743	0.4110
PAPPA	9	527.6214	0.4230

Thirteen genes (six lncRNAs and seven mRNAs) overlapped in each dimension.

The lncRNA we focused on are highlighted in bold.

### 3.3 RWR algorithm analysis of PTCRN

To prioritize PTC‐related genes, we performed an RWR algorithm analysis with PTCRN, using three validated PTC‐related genes as seed nodes. After the permutation test, lncRNA RP11‐159F24.1 was identified as a PTC‐related gene (*P* = 0.040). In the subnetwork, RP11‐159F24.1 could interact with 47 mRNAs through sponging has‐miR‐485‐5p (Figure 4).

### 3.4 Functional enrichment analyses of the mRNAs interacting with RP11‐159F24.1

**Figure 5:**
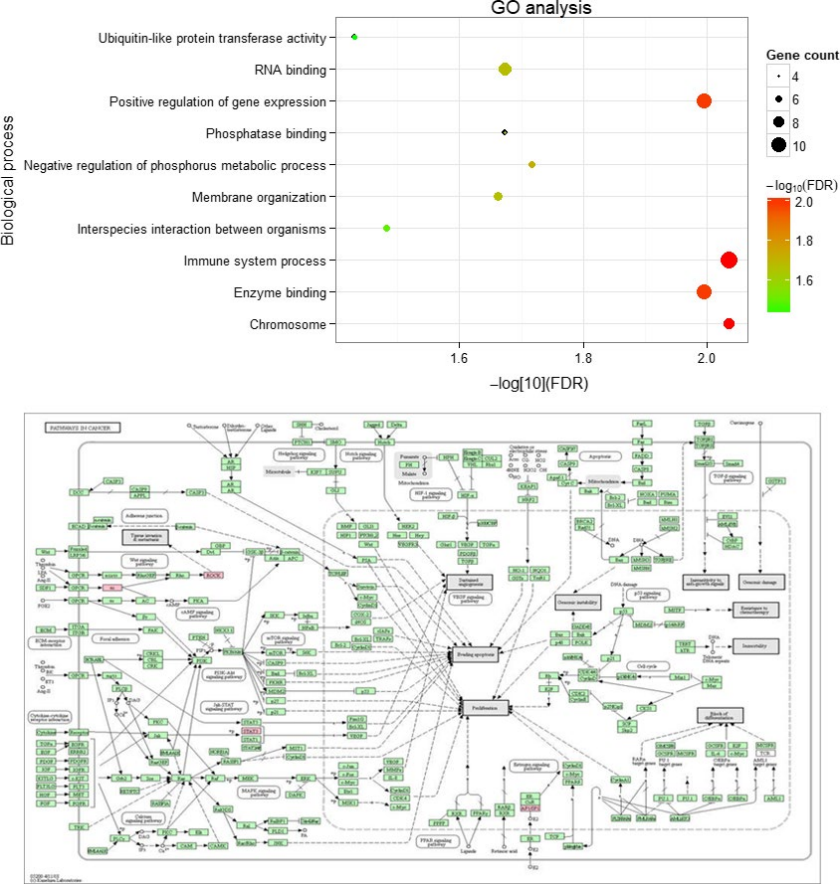
Gene ontology analysis and KEGG pathway analysis of the mRNAs interacting with lncRNA RP11‐159F24.1

In GO analysis, these 47 mRNAs were associated with multiple biological functions, like immune system process and gene expression regulation (FDR<0.05) (Figure 5). KEGG pathway analysis indicated these 47 mRNAs were involved into the pathways in cancer, especially ROCK, STAT3 and SP1.

### 3.5 Expression validation of RP11‐159F24.1 and its target miR‐485

**Figure 6:**
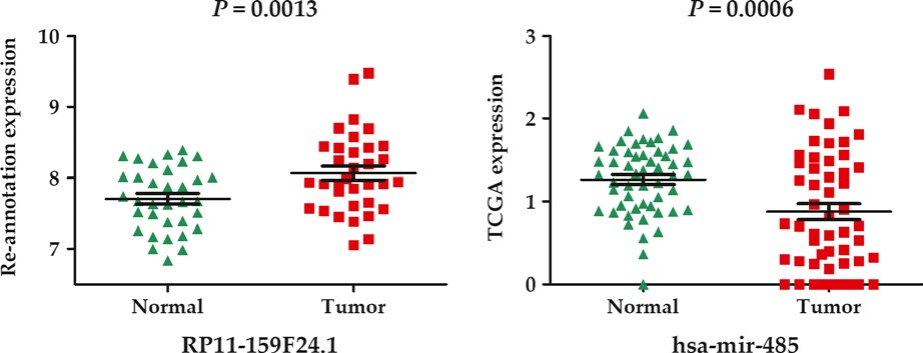
Expression validation of lncRNA RP11‐159F24.1 and its target miR‐485 in papillary thyroid cancer

Another GEO dataset of GSE35570 which contained 34 pairs of PTC and adjacent normal samples, was used to validate RP11‐159F24.1 expression. RP11‐159F24.1 also showed a significant higher expression level in PTC (*P* = 0.0013) (Figure 6). RNA‐sequencing data of 53 pairs samples indicated an obviously lower expression of miR‐485 in PTC (*P* = 0.0006).

## 4 DISCUSSION

In this study, we investigated the molecular mechanism of PTC by building a lncRNA‐miRNA‐mRNA regulatory network. First, the microarray expression profile was re‐annotated into a lncRNA‐mRNA biphasic profile. Then, lncRNA‐mRNA interactions were confirmed by established miRNA‐RNA data and hypergeometric test. Finally, a PTC‐related lncRNA‐miRNA‐mRNA network (PTCRN) was constructed by integrating differentially expressed genes with the RNA‐RNA networks.

To prioritize PTC‐related genes, we adopted two different methods to identify the hub nodes in PTCRN. First, topological analysis was conducted on the network, and the graph parameters indicated six lncRNAs and seven mRNAs as the hub nodes in the regulatory network. Among these genes, CAMTA1, CDC42 and RNF138 have been reported as oncogenes in multiple cancers,^17^, ^18^, ^19^ while CREBRF was a tumor suppressor in glioblastoma and a tumor promoter in gastric cancer.^20^, ^21^ Furthermore, CAMTA1 and CDC42 could interact with several lncRNAs to make significant effects on the tumorigenesis.^22^, ^23^

When performed RWR to the network, lncRNA RP11‐159F24.1 were also identified in association with the pathogenesis of PTC. In the subnetwork of RP11‐159F24.1, it could interact with 47 mRNAs though sponging the miRNA of has‐miR‐485‐5p. We supposed that the RP11‐159F24.1/miR‐485/mRNAs axis might play an import role in the development of PTC. As for RP11‐159F24.1, the validation set also indicated a higher expression in PTC. As the target of RP11‐159F24.1, miR‐485 showed a significantly lower expression in PTC, which was consistent with our hypothesis. miR‐485 (including miR‐485‐5p and miR‐485‐3p) has been reported as a tumor suppressor in multiple cancers.^24^, ^25^, ^26^ As the targets of miR‐485, the 47 mRNAs were found in association with several biological processes, especially pathways in cancer. Among these mRNAs, STAT3 and SP1 have been experimentally validated as a tumor promoter in PTC.^27^, ^28^ Thus, we thought the RP11‐159F24.1/miR‐485/mRNAs axis might play an important role in the pathogenesis of PTC.

Although we provided a novel approach to construct a PTC‐related lncRNA‐miRNA‐mRNA network and identify hub genes in the network, the limitations should be acknowledged for our study. As the probes were re‐annotated, the network could not cover all lncRNAs. We identified a total of 4232 lncRNAs, but still missed some popular lncRNAs, like ANCR, UCA1 and POU3F3.^29^, ^30^, ^31^ New complete expression profiles of lncRNAs, mRNAs and miRNAs were needed to construct a more comprehensive network and identify more hub genes.

In conclusion, this study has constructed a PTC‐related lncRNA‐miRNA‐mRNA network and identified the hub lncRNA RP11‐159F24.1 in the tumorigenesis, which provided novel insights to explore the underlying mechanism of PTC.

## CONFLICT OF INTEREST

None declared.
